# The role of sonication in developing synbiotic Beverages: A review

**DOI:** 10.1016/j.ultsonch.2024.106941

**Published:** 2024-06-08

**Authors:** Harsh B. Jadhav, Pintu Choudhary, Uday Annapure, Seema Ramniwas, Robert Mugabi, Gulzar Ahmad Nayik

**Affiliations:** a Department of Food Engineering and Technology, Institute of Chemical Technology, Matunga, Mumbai 400019, India; b PIHM, Unit UMET, INRAE, 369 Rue Jules Guesde 59650, Villeneuve d’Ascq -59650, France; c Department of Food Technology, CBL Government Polytechnic, Bhiwani, Haryana, India; d University Centre for Research and Development, Chandigarh University, Gharuan, Mohali 140413, Punjab, India; e Department of Food Technology and Nutrition, Makerere University, Kampala, Uganda; f Department of Food Science & Technology, Govt. Degree College, Shopian 192303, J&K, India

**Keywords:** Probiotic, Prebiotic, Synbiotic, Beverages, Ultrasound, Cavitation

## Abstract

Synbiotics are a combination of probiotic cells and prebiotic components and this harmonious association has numerous health benefits. Conventional processing technologies use high temperatures for processing which reduces the viability and the final quality of synbiotic beverages. Sonication is a rapidly growing technology in the food processing sector and can be employed for the formulation of synbiotic beverages with improved functionalities. The cavitation events generated during the sonication result in beneficial effects like increased viability of probiotic cells, enhanced bifidogenic characteristics of prebiotic components, less processing time, and high-quality products. The sonication process does not affect the sensory attributes of synbiotic beverages however, it alters the structure of prebiotics thus increasing the access by the probiotics. These positive effects are solely dependent on the type of ultrasound process and the ultrasound operating parameters. The review aims to provide information on the technological aspects of ultrasound, a brief about synbiotics, details on the ultrasound process used for the formulation of synbiotics, the influence of ultrasound operating parameters, and a focus on the research gap.

## Highlights

• Ultrasonication is a promising alternative to thermal processing for synbiotic beverages.
• Sonication enhances synbiotic beverages by improving probiotic viability and prebiotic functionality.
• Sonication preserves quality and sensory attributes better than high-temperature methods.
• Sonication reduces processing time and enhances beverage quality and functionality.

## 1 Introduction

Consumers have become more conscious about the food they are consuming, and the demand for health-friendly food has increased. The increasing demand has been the driving force for continuous innovation in the formulation of novel food products for the benefit of consumers. The desk-bound lifestyle and lack of physical exercise have resulted in increasing cases of chronic diseases across the globe and these diseases can be prevented by following a healthy diet [1]. Consuming food products containing functional components possesses numerous health benefits. The symbiotic food containing prebiotics and probiotics has many functional characteristics and benefits humans in many ways viz. preventing cancer growth, cardiovascular diseases, maintaining heart health, boosting the immune system, keeping the intestine healthy, etc. [2], [3]. The symbiotic foods are defenseless against the processing conditions and little alteration in the chemical or physical ways of processing can result in decreasing the population of probiotic bacteria in the symbiotic formulation [4].

The processing techniques used in the formulation of processed food products usually use higher temperature to reduce the load of pathogenic bacteria present in food. However, the use of higher temperature has a negative impact on the textural, sensory, and nutritional profile of food products, and in the case of symbiotic food, the higher temperature may result in reducing the population of beneficial microorganisms (probiotic) and can also result in structural deformation to prebiotic components [5], [6]. Regardless of the health benefits of such functional food products, consumer acceptance of these products is low because of the negative impact of thermal treatment on the quality of these products. Such losses in the quality of the synbiotic food material can be worked out by replacing thermal processing techniques with non-thermal technologies. As discussed, in synbiotic foods the higher temperature negatively affects both the prebiotic components and the probiotic bacteria, and for the higher survival rate of probiotic bacteria, the processing and storage temperature should be lower [7]. Non-thermal technologies have emerged as a novel and fast processing technologies that maintain quality of the processed food, among these non-thermal technologies, sonication is the rapidly growing technology that has been extensively researched for processing of food products [8], [9], [10].

**Fig. 1:**
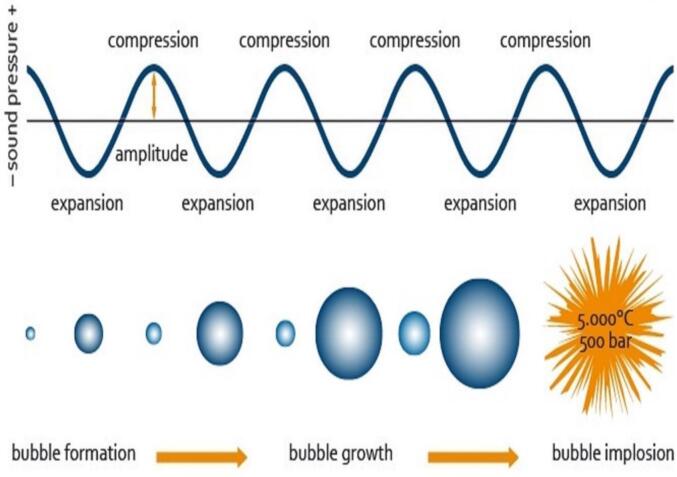
Formation, growth, and bursting of cavity bubble during the sonication process ([44]).

The power ultrasound with the frequency range of 20 – 25 kHz is usually preferred for processing of food. The sonication effect is observed due to the growth and sudden collapse of the cavity bubble (Fig. 1), the sound of sufficient frequency travels through the liquid food and forms cavities [11]. Due to the process of rectified diffusion, the formed cavities grow in size, and after attaining maximum size they collapse and release a huge amount of energy at the microscopic scale, this cavitation phenomenon is responsible for the beneficial effects on liquid food like thorough mixing, homogenization, degasification, forming stable emulsion, reduction in the pathogenic count, enhancing the availability of bioactive components, reducing the size of the fat globule, etc [12], [13], [14], [15], [16]. However, there is little information available on the sonication-assisted formulation of synbiotic beverages. The present review focuses on the sonication-assisted formulation of synbiotic beverages, technical details on sonication, the favorable sonication process like HIUO (High-intensity ultrasound operation) and LIUO (Low-intensity ultrasound operation), and further, the present scenario and the future foresight of sonication technology in the synbiotic food sector are also discussed in detail.

## 2 Synbiotics – A harmonious association

**Fig. 2:**
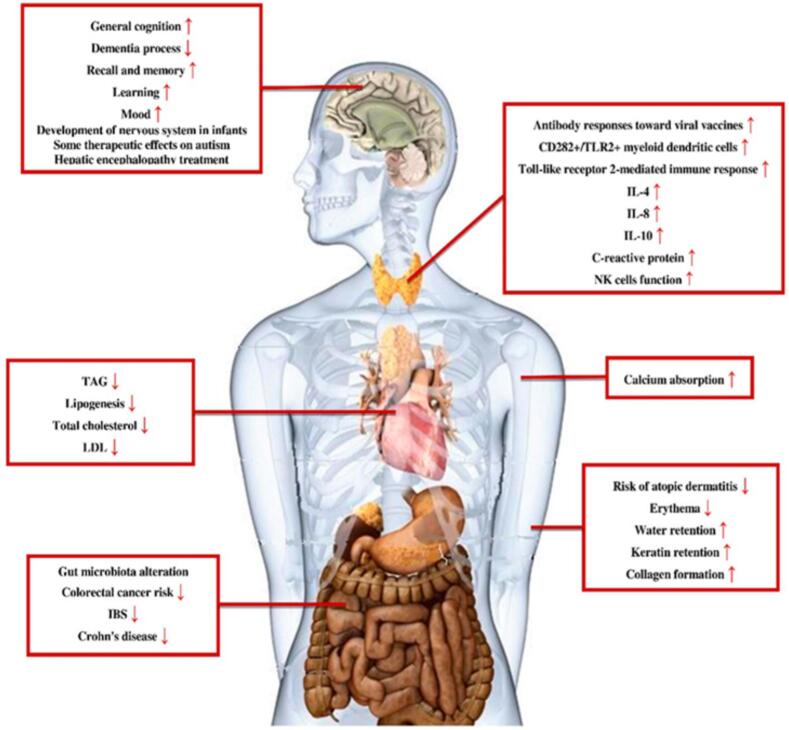
Beneficial effect of synbiotic on human health (Reproduced from Davani-Davari et al., (2019 [21])).

The primary objective of the nutrients consumed by humans is to provide the nutrition for physiological activities and overall growth. However, recently the focus of scientists has shifted towards the secondary role of nutrients, which includes the maintenance of human health by thwarting diseases. In the recent past, food containing beneficial bacteria and prebiotic components came into the limelight to redefine the consortium that exists between beneficial microorganisms and their positive effects on human beings. Synbiotics is the synergistic combination of the beneficial bacteria (probiotics) and substrate (prebiotic components) that has positive effects on the host. Prebiotic components have shorter chain lengths, low molecular weight, and are not digested in the human body [17]. However, these prebiotic components are utilized by the colonic microflora for their survival in the human gut. The harmonious association between prebiotics and probiotics generates metabolites that reduce the pH of the human gut, prevent the growth of pathogenic bacteria, and maintain human health [7], [18]. The positive association between the different combinations of probiotics and prebiotics is effective against health issues like cancer, diabetes, gastrointestinal problems, liver diseases, and respiratory problems [19]. The prebiotic components are selectively fermented by the probiotic microbes and the prebiotics serve as a primary source of nutrients for the growth and multiplication of probiotic bacteria. The concentration of metabolites like carbon disulfides, propionate, acetate, ketones, methyl acetate, etc increases in the gut and has a positive effect on human health [20]. The synergism also helps in reducing the accumulation of undesirable metabolites thereby reducing the chances of health issues [21]. A few of the prebiotic components that are commonly used in the formulation of synbiotic beverages are galactooligosaccharides, inulin, fructooligosaccharide, resistant starches, β-glucans, *trans*-galactooligosaccharides, fructans [22] along with the probiotic bacteria *Lactobacillu*s and *Bifidobacteria* sp. As discussed, the metabolites formed after the utilization of prebiotics by probiotics mainly include short-chain fatty acids and due to their smaller size, they can diffuse through the intestinal cells into the blood (Fig. 2). Along with the circulation of blood in the body, these metabolites can reach different organs and hence the synergism not only shows positive benefits on the gut but it also affects other organs positively [22], [23]. Breast milk is the best example of a synbiotic combination as it contains oligosaccharides (prebiotics) and lactic acid bacteria (probiotics) that help in the overall growth of infants and keep them healthy. The unripe bananas contain a higher proportion of resistant starches that have prebiotic characteristics and can be used in the formulation of synbiotic food products. For example, the addition of dried raw banana powder to fermented milk can result in the formulation of milk-based synbiotic beverages as reported by Batista et al. [24]. The synbiotic formulation showed higher viability of probiotic bacteria (more than 6 log CFU/g), showed an increase in the polyunsaturated fatty acids and the formation of components like ketones, carboxylic acid, and esters contributed to an improved flavor profile of the synbiotic milk-based fermented beverage. Hence, the demand for such products containing a combination of prebiotics and probiotics has increased as it not only helps in maintaining human health but also adds palatability to the food in which it is added. The conventional treatments, as discussed are not suitable for the production of synbiotic beverages as it has a negative effect on both the probiotic and prebiotic components hence it is important to understand the possible alternatives like sonication in the formation of synbiotic beverages to meet the growing demand from the consumers. Beneficial effect of synbiotic on human health is shown in Fig. 2.

## 3 Technological aspect of sonication

Sonication has been associated with the food processing sector and is being increasingly used for various applications like homogenization, degasification, foaming, drying, cutting, emulsification, freezing, and extraction of bioactive from the source [25]. The beneficial effect of this technology is achieved with the help of sound waves having a frequency higher than human hearing capacity (>16––20 kHz) and based on the operating frequencies the technology can be categorized into three major sections viz diagnostic, high frequency, and low frequency having working frequencies in the range of > 1 MHz, 20 kHz – 2 MHz and 20 kHz – 100 kHz respectively [26]. The practical application of this technology in the food sector uses low-frequency high-power (20–100 kHz, 10-1000Wcm^−1^) and high-frequency low-power (>100 kHz, <1Wcm^−1^) sonication. The sonication process using low frequency brings about chemical and physical alterations in the food product whereas the sonication with higher frequency is nondestructive and unable to generate the cavitation effect hence it is employed as an analytical and monitoring tool [27], [28]. During sonication, the cavitation effect is responsible for the intensification effect using low-frequency ultrasound. When the sound wave travels through the liquid food medium, it undergoes alternating compression and expansion cycles. During these cycles, the cavity bubbles are formed which grow in size and attain a maximum size beyond which the bubbles are incapable of absorbing additional energy and at this point, the cavity bubble implodes violently. The violent collapse of the cavity bubble increases pressure and temperature and brings about a sterilization effect in liquid food, a decrease in the microbial load due to damage caused to the cells of microbes. The cell wall of the Gram-positive bacteria is thicker and tougher than the cell wall of Gram-negative bacteria, hence the Gram-negative bacteria can be easily inactivated using sonication as compared with the Gram-positive bacteria [27]. Similarly, bacterial spores, fungi, cocci, and anaerobic microbes show higher resistance towards sonication a s compared with the vegetative cells, anaerobic microbes, and bacilli [29]. However, in the case of formulation of synbiotic beverages using sonication, the cavitation effect should not pose any harm to the growth of probiotic bacteria. This can be achieved by close monitoring of the ultrasound operating parameters and any fluctuations in these parameters may result in decreasing the probiotic concentration beyond the acceptable level in synbiotic beverages.

### 3.1 Sonication processes

**Fig. 3:**
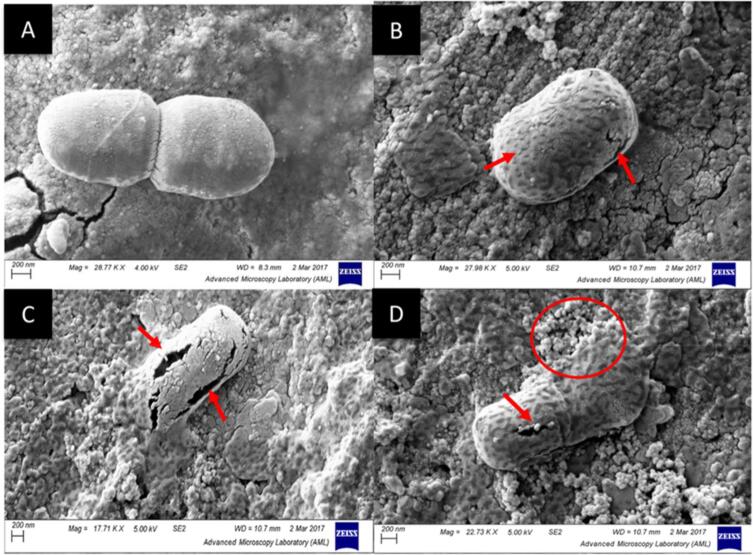
SEM images showing sonoporation due to ultrasonication (a) untreated sample, (b-d) treated sample showing cell rupture and voids after exposure to ultrasound treatment of frequency 20 kHz (Reproduced from Ojha et al., (2018)[26].

Sonication has been found to have both positive and negative effects on the growth of microorganisms. The exposure of the cell membrane to the ultrasound results in increasing cell permeability usually referred to as sonoporation. Sonoporation forms smaller voids (Fig. 3) on the cell membranes of microbes helping in the removal of undesirable and toxic substances and also helps in the transfer of nutrients across cells. As a result, cellular functions like growth, transport of nutrients, multiplication, and enzyme activities are affected. The higher degree of sonoporation is undesirable as it causes leakage of cell content due to damage to the cellular membrane caused by cavitation events. Unlike this, the lower degree of sonoporation is always desirable as it enhances mass transfer across cells/membranes, helps in the removal of toxic substances from microbial cells, and increases nutrient and oxygen supply to the cell membrane [30]. To better understand the effect of ultrasound operating parameters, the two operations i.e., LIUO and HIUO are discussed. The HIUO enhances the viability of probiotic cells and increases their activity resulting in higher utilization of prebiotic components and lowering the fermentation time. The high-intensity operations also enhance the transgalactosylation of probiotic cells and the hydrolysis of disaccharides like lactose. The sonication process enables the release of enzymes from the cell and increases their activity. For example the release of enzymes like proteases, and β-galactosidase due to sonoporation results in increased hydrolysis of protein and sugar that increases the growth of probiotic cells [31]. One of the major challenges in synbiotic beverages is the use of prebiotic components which may cause instability in the final product, and this can be overcome by the application of sonication. The HIUO does not bring about any structural deformation in the prebiotic component but can sometimes result in hydrolysis of the prebiotic component [32], [33]. In the case of synbiotic beverages, a low degree of hydrolysis could be beneficial as it enhances the bifidogenic characteristics of prebiotics. But there are certain studies reported in literature, where the HIUO has caused structural modifications in the prebiotic components. This can also be considered a positive point as the structural modification of the prebiotic component can result in the release of simple components due to the breakage of the polymer molecule and these simple molecules can be easily utilized by the probiotic cells for their growth and development in the gut [34], [35].

The LIUO is also one of the fastest-growing sonication technologies for the processing of functional beverages involving probiotics and prebiotic components. The beneficial effects of LIUO accelerate the growth of probiotics present in the synbiotic formulation, promote the fermentation process, increase the formation of metabolites, and activate enzymes [36]. However, LIUO is usually preferred for non-destructive operations like monitoring enzymatic reactions, growth of microbes, gelling process, and determining the quality of the product (texture, rheology, maturity) [37]. A study by H. Wang et al., (2021) evaluated the effect of LIUO on the *Lactobacillus plantarum* growth stages. The authors described the formulation of fruit beverages by the addition of *Lactobacillus plantarum* with the application of LIUO having energy densities of 58.3 W/L and 93.6 W/L. The author concluded that the LIUO enhances the growth of bacteria was enhanced by 0.51log CFU/mL at the lag phase and 0.31log CFU/mL at the logarithmic phase [38]. Another study by Niamah, (2019) also investigated the effect of LIUO on the growth of probiotic bacteria. The author studied the effect of 40 kHz frequency and irradiation time 0 – 20 min on the growth of probiotic *Lactobacillus* and *Bifidobacterium* sp. The author concluded that the LIUO showed beneficial effects on the growth of probiotic bacteria and release of β-galactosidase till 10 min of irradiation time [39]. The optimized LIUO parameters for the higher probiotic growth were 40 kHz frequency, 10 min irradiation time, and 116 W power. However, there are few studies reported in the literature involving the use of low-intensity ultrasound in the formulation of synbiotic beverages. Future research should focus on the use of different combinations of probiotic cultures and prebiotic components in the formulation of synbiotic beverages. This will help in exploring the technology for synbiotic products and will further provide a way to commercialize the process for large-scale production of synbiotic beverages with an application of ultrasound.

### 3.2 Sonication parameters

The ultrasound operating parameters are responsible for the quality of final synbiotic beverages in terms of the survival of probiotic bacteria, prebiotics, physicochemical characteristics, and sensory profile. Operating parameters like power and irradiation time play key roles in the development of synbiotic beverages. Power in the sonication process is an important parameter that is influenced by the wave amplitude and can be varied by varying the applied voltage. Generally, increasing the power of the ultrasound-assisted process gives more intensification benefits, but these benefits can be seen up to an optimum level, and beyond this level, the positive effect is minimized and food is negatively affected. An increase in the power beyond the optimum level results in the occurrence of excessive cavitation events giving rise to the cushioning effect. The study on the effect of ultrasound power on the formulation of soymilk-based synbiotic beverages was reported by Yeo & Liong [40]. The authors investigated the effect of 20 W, 60 W, and 100 W on the characteristics of synbiotic beverages. The viability of *Lactobacillus sp*2113 and *B.longum* 8643 increased with an increase in the power up to 60 W and a further increase in power from 60 to 100 W resulted in decreasing their viability from 6.72 to 6.26 log_10_ CFU/mL and 6.43 to 6.36 log_10_ CFU/mL respectively after treatment. However, the treatment at 100 W showed a positive effect on the activity of enzymes, the β-glucosidase showed increased activity which helped in a higher rate of formation of aglycones from β-glucosides. The effect of power also depends on the type of probiotics present in the synbiotic formulation, the probiotics with tough and rigid cell walls will be less affected and can survive in high-power intensities, as compared with the probiotics having thin cell membranes where rupturing of cell and leakage of cellular components can lead to death at high power intensities. Like power, the other sonication parameters also influence the survival of probiotic bacteria in the synbiotic beverages. The health benefits of the synbiotic formulation are best enjoyed if it contains the minimum recommended load of probiotics and minimum recommended concentration of prebiotics. The frequency of the ultrasound process is not much varied in the food processing operation, as most of the food processing operations are carried out in the frequency range of 20 – 25 kHz [41].

The other important parameter is irradiation time, the increase in the irradiation time, increases the exposure of probiotic cells and prebiotic components to ultrasound and intensifies the process in terms of increase in viability, low degree sonoporation, higher rate of formation of metabolites, that in turn helps in maintaining the health-friendly characteristics of the synbiotic beverages. However, continuous increase in the irradiation time increases exposure to ultrasound beyond the tolerable limits leading to a decrease in the positive effects and an increase in the negative impact on the synbiotic beverages. The higher exposure time can result in a higher degree of sonoporation leading to loss of cellular components, which can cause irreversible structural changes in the prebiotic components thus making it unavailable for the probiotic bacteria to utilize and grow. Apart from this, the higher exposure time can also deteriorate the other biological macromolecules present in the synbiotic beverages. In a study involving the formulation of synbiotic beverages, the irradiation time was varied as 60, 120, and 180. The viability of *L. fermentum, L. acidophilus* FTDC, and *L. gasseri decreased* with an increase in the exposure time from 120 s to 180 s from 7.03, 7.30, 7.24 log CFU/mL to 6.82, 7.25, 7.19 log CFU/mL respectively. Additionally, the increase in exposure time also triggered lipid oxidation as detected by an increase in the malondialdehyde concentration in the synbiotic beverage, this affects the sensory profile of the synbiotic beverages [42]. The higher exposure time also reduces post-acidification in the products like yogurts, due to reduced viability of the probiotic cells [43]. It is difficult to draw an exact idea of the optimized ultrasound parameters for the formulation of synbiotic beverages due to limited studies reported in the literature. The topic provides an immense scope to explore the efficacy of the ultrasound process in the formulation of synbiotic beverages. The literature also lacks studies showing the potential positive and negative effects of sonication on the prebiotic components present in synbiotic beverages.

## 4 Concluding remark and research gap

The growing health concern has triggered the need for functional food like synbiotics which have numerous health benefits. However, the benefits of synbiotics can only be enjoyed, if they contain a minimum load of probiotic bacteria and the prebiotic components. The thermal processing exposes synbiotic beverages to a higher temperature which causes a significant reduction in the viability of probiotic bacteria and also reduces the quality of the final product. The continuous improvement in technology gave a solution to this in the form of non-thermal processing. Sonication is one of the most widely accepted and practiced technologies in the food processing sector and it is emerging as a probable solution to the thermal processing of synbiotic beverages. Sonication is found to have a beneficial effect on both the probiotic and prebiotic components present in the synbiotic beverages. The process helps increase the viability of the probiotic cells, increase the activity of probiotics, lower the degree of sonoporation, increase the supply of essential nutrients and oxygen to the probiotic cell, causes a small degree of hydrolysis in prebiotic components that enhance the bifidogenic characteristics of this components and increases the metabolites like short chain fatty acids which has a positive effect on human health. Two sonication processes i.e. HIUO and LIUO are employed for processing, among these two processes HIUO is used commonly as it brings about significant physical and chemical changes in the food but some studies have also used LIUO for the formulation of synbiotic beverages and reported promising results. Ultrasound parameters like power and irradiation time play a key role in transferring the beneficial effect of this technology in the processing of synbiotic beverages. The high power and long exposure time result in reversing the positive effect of this technology and adversely affecting the viability of probiotic cells and structural deformation in prebiotic components. However, the use of ultrasound in the formulation of synbiotic beverages is in its infancy and a lot of future research needs to be undertaken to understand the actual mechanism. The literature has reported very few studies in which ultrasound is used for synbiotic beverages, hence it is difficult to arrive at a concluding remark on the optimized ultrasound parameters for the formulation of high-quality synbiotic beverages. In the future, the work should focus on the use of different combinations of prebiotics and probiotics in the formulation of dairy and non-dairy synbiotic beverages with much focus on optimization of all the ultrasound parameters like power, frequency, duty cycle, irradiation time, etc. Further research in this area will help in commercializing the synbiotic beverages in the market across the globe and will help to formulate high-quality products that will have higher consumer acceptance.

## CRediT authorship contribution statement

**Harsh B. Jadhav:** Writing – review & editing, Writing – original draft, Visualization, Investigation, Data curation, Conceptualization. **Pintu Choudhary:** Writing – review & editing, Supervision, Software, Project administration, Methodology, Funding acquisition, Conceptualization. **Uday Annapure:** Writing – review & editing, Writing – original draft, Supervision, Resources, Funding acquisition, Data curation, Conceptualization. **Seema Ramniwas:** Writing – review & editing, Writing – original draft, Validation, Supervision, Funding acquisition, Conceptualization. **Robert Mugabi:** Writing – review & editing, Writing – original draft, Software, Resources, Methodology, Funding acquisition, Data curation. **Gulzar Ahmad Nayik:** Writing – review & editing, Writing – original draft, Validation, Supervision, Formal analysis, Conceptualization.

## Declaration of competing interest

The authors declare that they have no known competing financial interests or personal relationships that could have appeared to influence the work reported in this paper.

## Data Availability

Data will be made available on request.
